# A genetic algorithm for optimal assembly of pairwise forced-choice questionnaires

**DOI:** 10.3758/s13428-021-01677-4

**Published:** 2021-09-09

**Authors:** Rodrigo Schames Kreitchmann, Francisco J. Abad, Miguel A. Sorrel

**Affiliations:** Department of Social Psychology and Methodology, Faculty of Psychology, Universidad Autónoma de Madrid, Madrid, Calle Iván Pavlov, 6, Ciudad Universitaria de Cantoblanco, 28049 Madrid, Spain

**Keywords:** forced-choice format, ipsative data, multidimensional item response theory, reliability, test assembly, genetic algorithms

## Abstract

The use of multidimensional forced-choice questionnaires has been proposed as a means of improving validity in the assessment of non-cognitive attributes in high-stakes scenarios. However, the reduced precision of trait estimates in this questionnaire format is an important drawback. Accordingly, this article presents an optimization procedure for assembling pairwise forced-choice questionnaires while maximizing posterior marginal reliabilities. This procedure is performed through the adaptation of a known genetic algorithm (GA) for combinatorial problems. In a simulation study, the efficiency of the proposed procedure was compared with a quasi-brute-force (BF) search. For this purpose, five-dimensional item pools were simulated to emulate the real problem of generating a forced-choice personality questionnaire under the five-factor model. Three factors were manipulated: (1) the length of the questionnaire, (2) the relative item pool size with respect to the questionnaire’s length, and (3) the true correlations between traits. The recovery of the person parameters for each assembled questionnaire was evaluated through the squared correlation between estimated and true parameters, the root mean square error between the estimated and true parameters, the average difference between the estimated and true inter-trait correlations, and the average standard error for each trait level. The proposed GA offered more accurate trait estimates than the BF search within a reasonable computation time in every simulation condition. Such improvements were especially important when measuring correlated traits and when the relative item pool sizes were higher. A user-friendly online implementation of the algorithm was made available to the users.

Several meta-analytic studies from the last decades indicate that non-cognitive domains such as personality, motivation, and leadership can offer predictive power over academic and work performance (e.g., Judge et al., 2013; Montano et al., 2017; Poropat, 2009; Richardson et al., 2012). Such findings have increased interest in the structured assessment of these characteristics for selection purposes (Salgado & De Fruyt, 2017). These non-cognitive dimensions have been traditionally measured using rating scale-based self-reports, in which respondents must indicate their agreement with a set of statements describing some behaviors (e.g., Likert scales). However, this assessment format in the selection scenario has been shown to be susceptible to important response biases such as acquiescence (ACQ), social desirability responding (SDR), and faking (e.g., Heggestad et al., 2006; Paulhus, 1991). The ACQ consists of a tendency to respond toward the upper end of the rating scale, regardless of one’s true trait level. In turn, SDR implies that respondents have a tendency to provide overly positive self-descriptions, either caused by self-deception or impression management (Paulhus, 2002). Finally, faking refers to a situational rather than a general tendency and the intentional behavior of misrepresenting oneself to achieve personal goals, such as being selected for a job (MacCann et al., 2011).

If unaccounted for, such response biases may affect the fairness and validity of the assessments, compromising the selection results. For instance, candidates with optimum levels in the constructs of interest who do not engage in SDR or faking can score lower than less appropriate candidates that respond in a more desirable way. Additionally, the presence of the ACQ style has been found to be directly associated with age and inversely related to years of formal education (e.g., Weijters et al., 2010), which can also affect the selection process. Furthermore, these response styles may distort the questionnaire’s psychometric properties. For example, the existence of response biases may bias the item intercorrelation matrix, distorting the questionnaire’s estimated factor structure and leading to model misfit (e.g., Abad et al., 2018; Navarro-González et al., 2016). In addition, SDR and ACQ may inflate the reliability estimates and convergent validities with other rating scale-based measures due to the common variance introduced by these response styles (e.g., Soto et al., 2008; Soto & John, 2019), giving the impression that an assessment is more trustworthy than it truly is.

The use of multidimensional forced-choice questionnaires (FCQs) has been proposed to prevent these response biases in the assessment of non-cognitive domains (e.g., Cao & Drasgow, 2019; Cheung & Chan, 2002; Salgado & Táuriz, 2014; Wetzel et al., 2021), as they offer comparable or better convergent and criterion-related validity (e.g., Kreitchmann et al., 2019; Otero et al., 2020). This format differs from rating scales, in that instead of indicating one’s agreement with a statement on an ordinal scale, respondents must rank two or more statements within a block according to their agreement with each statement. On the one hand, by dispensing the ordinal scale, FCQ eliminates acquiescent responding (Cheung & Chan, 2002; Ferrando et al., 2011). On the other hand, if statements have similar social desirability, SDR and faking will be harder to engage with (Lee & Joo, 2021; Wetzel et al., 2021).

As is widely known, an assessment’s reliability sets the upper boundary for other aspects of validity. Thus, attenuating the effects of response styles with forced-choice formats will only be truly effective if the questionnaire is able to provide accurate scores. As will be detailed further, a single pool of items can lead to FCQs with very different reliabilities, depending on the specific characteristics of the items forming each block. Unfortunately, given these questionnaire’s high dimensionality and the complexity of the combinatorics for assembling items in blocks, the existing methods to maximize test reliabilities with single-statement items, such as linear programming, are not feasible for FCQ. Specifically, the number of possible combinations of *N* items in *J* blocks of size *V* is:

1 $$ \frac{N!}{J!\left(N- JV\right)!V{!}^J}, $$

which will most likely be a large number under realistic conditions. For instance, the simple assembly of 30 forced-choice pairs out of 60 items (i.e., *J* = 30, *V* = 2, *N* = 60) derives into approximately 2.92 × 10^40^ unique candidate questionnaires. Thus, even in a very optimistic scenario in which forming and evaluating a questionnaire takes a nanosecond, it would take longer than the age of the universe to consider all possibilities. Currently, no tool is available to efficiently address this problem. Therefore, this article aims to offer a procedure capable of examining this search space and optimizing the accuracy of attribute scores. For simplicity, this article focuses on the assembly of forced-choice pairs (i.e., *V* = 2), although the procedure presented here can be further extended for greater *V* values.

## Forced-choice modeling

Historically, forced-choice-based measurement has been called *ipsative* to denote an interdependency among the trait scores as a result of the forced-choice format, since scoring higher in one dimension necessarily implies scoring lower in the other dimensions presented in the same blocks. In this sense, under classical test theory, *ipsative* measures can only be compared within each subject (Cattell, 1944). As a result, validity evidence for *ipsative* measures would also be impaired. Specifically, the expected intercorrelation between scores in completely *ipsative* measurements would be necessarily negative, that is, −1/(*D* − 1), where *D* is the number of traits evaluated, while the sum of their correlations with each external criterion would be zero (Hicks, 1970).

Recent research has shown that score *ipsativity* is not a consequence of the response format itself, but rather of the inadequate modeling of the psychological process underlying comparative judgments (Meade, 2004). Currently, there are a wide variety of models under confirmatory factor analysis and item response theory (IRT) that enable us to outline the response processes involved in forced-choice formats and to obtain normative scores (Brown & Maydeu-Olivares, 2011; Bunji & Okada, 2020; McCloy et al., 2005; Morillo et al., 2016; Stark et al., 2005). The multi-unidimensional pairwise preference (MUPP; Stark et al., 2005) framework for forced-choice pairs, for instance, conceives the response process as a result of independent evaluation of agreement with each statement in a pair and the further decision of which to select. Equation 2 provides the probability of endorsing one statement over the other:

2 $$ P\left({y}_{i,j}=1\right)=\frac{P\left({x}_{i,{j}_1}=1\right)P\left({x}_{i,{j}_2}=0\right)}{P\left({x}_{i,{j}_1}=1\right)P\left({x}_{i,{j}_2}=0\right)+P\left({x}_{i,{j}_1}=0\right)P\left({x}_{i,{j}_2}=1\right)}, $$

where *y*_*i*, *j*_ denotes the position of the selected item on the block (i.e., 1 or 2), and ${x}_{i,{j}_1}$ and ${x}_{i,{j}_2}$ are the latent responses of subject *i* for items *j*_1_ and *j*_2_, respectively, being equal to 1 if respondent *i* endorses the item, and 0 otherwise. Please note that the model makes no provisions for the endorsement of both or none of the statements; therefore, it assumes that respondents in these situations must reevaluate each statement independently until a preference is found (Stark et al., 2005).

Within the MUPP framework, the model underlying a subject’s probability of agreement with each statement, *P*(*x*_*i*, *j*_), can be defined either from a dominance perspective (i.e., the probability of agreement increases monotonically with trait level) or from an ideal-point understanding (i.e., the probability of agreement is non-monotonic and increases as trait level and item threshold approach). Although the appropriateness of these models relies mainly on empirical grounds, currently most items are dominance items (Brown & Maydeu-Olivares, 2010), and therefore, in this study, we will only address the MUPP’s dominance variant.

By assuming a two-parameter logistic (2PL), the probability of agreement with each *p*^th^ statement in the *j*^th^ pair conditioned on the *i*^th^ person’s true level in the *d*^th^ latent trait (${\theta}_{i,{d}_{j_p}}$) is given by:

3 $$ P\left({x}_{i,{j}_p}=1|{\theta}_{i,{d}_{j_p}}\right)=\frac{\exp \left({a}_{j_p}{\theta}_{i,{d}_{j_p}}+{c}_{j_p}\right)}{1+\exp \left({a}_{j_p}{\theta}_{i,{d}_{j_p}}+{c}_{j_p}\right)}, $$

where ${a}_{j_p}$and ${c}_{j_p}$ are the slope and intercept parameters, respectively, with ${c}_{j_p}=-{a}_{j_p}{b}_{j_p}$, in which ${b}_{j_p}$ is item difficulty in the traditional IRT parameterization.

By replacing the $\mathrm{P}\left({x}_{i,{j}_p}\right)$ terms from the general MUPP model using Eq. 3, the products in Eq. 2 are:

4 $$ {\displaystyle \begin{array}{l}P\left({x}_{i,{j}_1}=1|{\theta}_{i,{d}_{j_1}}\right)P\left({x}_{i,{j}_2}=0|{\theta}_{i,{d}_{j_2}}\right)=\frac{\exp \left[{a}_{j_1}{\theta}_{i,{d}_{j_1}}+{c}_{j_1}\right]}{1+\exp \left[{a}_{j_1}{\theta}_{i,{d}_{j_1}}+{c}_{j_1}\right]}\frac{1}{1+\exp \left[{a}_{j_2}{\theta}_{i,{d}_{j_2}}+{c}_{j_2}\right]}\\ {}\mathrm{and}\\ {}P\left({x}_{i,{j}_1}=0|{\theta}_{i,{d}_{j_1}}\right)P\left({x}_{i,{j}_2}=1|{\theta}_{i,{d}_{j_2}}\right)=\frac{1}{1+\exp \left[{a}_{j_1}{\theta}_{i,{d}_{j_1}}+{c}_{j_1}\right]}\frac{\exp \left[{a}_{j_2}{\theta}_{i,{d}_{j_2}}+{c}_{j_2}\right]}{1+\exp \left[{a}_{j_2}{\theta}_{i,{d}_{j_2}}+{c}_{j_2}\right]}.\end{array}} $$

Therefore, Eq. 2 can be simplified to the MUPP-2PL model (Morillo et al., 2016):

5 $$ P\left({y}_{i,j}=1|{\theta}_{i,{d}_{j_1}},{\theta}_{i,{d}_{j_2}}\right)=\frac{\exp \left[{a}_{j_1}{\theta}_{i,{d}_{j_1}}-{a}_{j_2}{\theta}_{i,{d}_{j_2}}+\left({c}_{j_1}-{c}_{j_2}\right)\right]}{1+\exp \left[{a}_{j_1}{\theta}_{i,{d}_{j_1}}-{a}_{j_2}{\theta}_{i,{d}_{j_2}}+\left({c}_{j_1}-{c}_{j_2}\right)\right]} $$

which, for *D* dimensions, can be parameterized as:

6 $$ P\left({y}_{i,j}=1|{\boldsymbol{\theta}}_{\boldsymbol{i}}\right)=\frac{\exp \left({\mathbf{s}}_{\boldsymbol{j}}^{\prime }{\boldsymbol{\uptheta}}_{\boldsymbol{i}}+{c}_j\right)}{1+\exp \left({\mathbf{s}}_{\boldsymbol{j}}^{\prime }{\boldsymbol{\uptheta}}_{\boldsymbol{i}}+{c}_j\right)}, $$

where **θ**_*i*_ is a *D* × 1 vector containing the trait level scores of the *i*^th^ subject, and **s'**_**j**_ is a 1 × *D* vector including the scale parameters for the *D* measured dimensions, where *s*_*j*, *d*_ = 0 if the items do not measure the dimension *d,* and ${s}_{j,d}={p}_{j_p}{a}_{j_p}$, being ${p}_{j_p}$ = +1 or ${p}_{j_p}$ = −1 depending on the position of the item measuring the dimension *d* on the block (i.e., first or second, respectively). Please note that this definition of ${p}_{j_p}$ is adequate if the data are encoded as *y*_*i*, *j*_ = 1 and *y*_*i*, *j*_ = 2 for endorsement of the first and second statements, respectively; otherwise, it will provide inverted trait estimates. Given the previous notation, parameter *c*_*j*_ represents the block threshold, where ${c}_j={c}_{j_1}-{c}_{j_2}$. As shown in Eq. 6, the MUPP-2PL response function is identical to the multidimensional compensatory logistic model (MCLM; McKinley & Reckase, 1982), with the exception that for modeling endorsement of the first item in a block (*y*_*i*, *j*_ = 1), the scale parameter of the second item will be the negative of the original item discrimination parameter under the 2PL.

The accuracy of the maximum-likelihood estimates of the scores can be approximated through the asymptotic variances of the trait estimators obtained from the diagonal of the inverse of the Fisher test information function (TIF). In turn, the Fisher information function at the block and questionnaire levels under the MUPP-2PL can be defined as in Eqs. 7 and 8, respectively, where *Q*_*j*_(**θ**) = 1 − *P*_*j*_(**θ**). Note that Eq. 8 assumes conditional independence between blocks; thus, each item must not be included in more than one block.

7 $$ {\mathbf{I}}_j\left(\boldsymbol{\uptheta} \right)={\mathbf{s}}_j{\mathbf{s}}_j^{\prime }{P}_j\left(\boldsymbol{\uptheta} \right){Q}_j\left(\boldsymbol{\uptheta} \right), $$

8 $$ \mathbf{I}\left(\boldsymbol{\uptheta} \right)=\sum \limits_{j=1}^J{I}_j\left(\boldsymbol{\uptheta} \right). $$

As can be seen in Eqs. 7 and 8, the asymptotic variances of the **θ** estimators depend on (1) the product of the scale parameters in each block and (2) the product of the MUPP-2PL response probabilities, conditional to **θ**, for either item in the block.

It has been found that regardless of each block’s individual characteristics, some questionnaire conditions can still lead to some degree of *ipsativity*, which may undermine the precision of the normative scores and offer negatively biased trait intercorrelations. On the one hand, under the dominance framework, the **S** matrix (a *J* × *D* matrix with the **s** vectors for every block as defined in Eq. 6) should be of full rank in order for the model to be identified. This condition will normally be met unless the scale parameters have special properties, for example, if all scale parameters are equal within every block or within every dimension (Brown, 2016). On the other hand, some aspects of questionnaire design have been found to improve the precision of trait estimates. For instance, under ideal-point IRT models, Stark et al. (2005) indicate the necessity of including unidimensional blocks to help identify the metric of the estimates. In addition, Brown and Maydeu-Olivares (2011, 2018) provide some general guidelines for constructing questionnaires under dominance IRT models. Specifically, these authors outline the positive effect of the following aspects on estimation precision: (1) the inclusion of blocks of items with different keyed directions, (2) the assessment of a large number of traits, (3) a low average correlation between traits, and (4) the increase in the number of statements forming each block. Regarding the latter, blocks of three and four items were found to provide higher reliability than pairs (e.g., Brown & Maydeu-Olivares, 2011; Joo et al., 2020).

As pointed out, the inclusion of blocks composed of items keyed in different directions (i.e., different polarities) is effective for improving estimation accuracy. However, researchers argue that hetero-polar blocks can be problematic in practice (Bürkner et al., 2019; Lee & Joo, 2021; Morillo et al., 2016). In this sense, Bürkner et al. (2019) outline four main reasons for not using unequally keyed blocks. First, judging one’s agreement with negatively keyed items can be cognitively demanding, compounded with the fact that the forced-choice format itself is already somewhat challenging (Sass et al., 2020), may affect the response process and compromise the construct validity. Second, negatively keyed items may add methodological variance (Dueber et al., 2019), forming a separate method factor. Third, if traits are oriented in the same direction as social desirability, positively keyed items will most probably be socially desirable, whereas negatively keyed items will be undesirable, and unequally keyed blocks will have a clearly more socially desirable option. Therefore, hetero-polar blocks may fail to control social desirability biases, which is one of the main merits of forced-choice formats. Fourth, and finally, in realistic scenarios, if respondents are able to identify and select the most desirable option in a block, that block will be uninformative for person parameter estimation (Wang et al., 2017) and may not improve the accuracy of trait estimates as expected.

Although several authors have raised the question of whether blocks with opposite-keyed items are robust to faking (e.g., Bürkner et al., 2019; Lee & Joo, 2021; Ng et al., 2021), there is still no empirical investigation directly comparing homo-polar (i.e., same polarities) and hetero-polar blocks with normative scoring. On the one hand, as evidence for the inclusion of hetero-polar blocks, Wetzel et al. (2021) found that FCQs with hetero-polar blocks were still more robust to faking than single-stimulus items (i.e., rating scales). On the other hand, Lee and Joo (2021) analyzed the invariance of item parameters in honest and faking conditions and suggested that hetero-polar blocks may be less invariant than homo-polar blocks. In addition, in counterpoint to Bürkner et al.’s (2019) first argument, the cognitive response process underlying negatively keyed items in forced-choice blocks has not yet been empirically investigated. Regarding Bürkner et al.’s (2019) second point, although a separate method factor for negatively keyed items may be expected for single-stimulus responses, we agree with one of the reviewer’s suggestion that it may be associated with acquiescence bias and might not be generalized for the forced-choice format. Finally, it is the opinion of the authors of this article that this debate and future investigations on the subject should be defined in more specific terms. For instance, it can be hypothesized that the inclusion of opposite-keyed item blocks in low-stakes scenarios may improve the accuracy of trait estimates with little harm to the validity of the assessment due to self-deception. In addition, in high-stakes scenarios, it can be postulated that if the traits being compared within a block have neutral social desirability, the inclusion of negatively keyed items may not affect the validity of the measurement of such traits. However, the inclusion of hetero-polar blocks has yet to be clarified through empirical studies. Therefore, in this study, two scenarios were considered. First, FCQ optimization was investigated using only positively keyed items. Later, a follow-up study is presented, including both positively keyed and opposite-keyed item pairs.

## Overview of test optimization

The IRT constitutes the perfect framework for test assembly with the goal of maximizing precision on certain pre-specified trait levels. For unidimensional models, under the assumption of local independence between the items, the TIF, *I*(*θ*), reflects the sum of the item information functions and is asymptotic to the variance of the maximum-likelihood estimator of *θ*. This aggregation principle allows us to conceptualize the assembly as a constrained combinatorial linear optimization problem, in which the inclusion of the items in the test is modeled as a vector **z** of binary decision variables (0: non-selected; 1: selected), aiming to maximize the desired objective function (e.g., the test information for a specific *θ* value). For instance, searching for items that minimize $\operatorname{var}\left(\hat{\theta}|\theta \right)$ is asymptotically equivalent to maximizing:

9 $$ \sum \limits_j{z}_j{I}_j\left(\theta \right). $$

These types of optimization problems, with the possibility of adding additional restrictions such as test length and word count, can be solved using mixed-integer programming (MIP). However, test assembly becomes more complicated as the dimensionality of the questionnaires increases, as in FCQ, because the TIF becomes an information matrix (see Eq. 8). For instance, for two-dimensional questionnaires, the TIF is given by:

10 $$ \mathbf{I}\left(\boldsymbol{\uptheta} \right)=\left[\begin{array}{cc}\sum \limits_j{s}_{j,1}^2{P}_j\left(\boldsymbol{\uptheta} \right){Q}_j\left(\boldsymbol{\uptheta} \right)& \sum \limits_j{s}_{j,1}{s}_{j,2}{P}_j\left(\boldsymbol{\uptheta} \right){Q}_j\left(\boldsymbol{\uptheta} \right)\\ {}\sum \limits_j{s}_{j,1}{s}_{j,2}{P}_j\left(\boldsymbol{\uptheta} \right){Q}_j\left(\boldsymbol{\uptheta} \right)& \sum \limits_j{s}_{j,2}^2{P}_j\left(\boldsymbol{\uptheta} \right){Q}_j\left(\boldsymbol{\uptheta} \right)\end{array}\right]. $$

The asymptotic trait estimator variance becomes (van der Linden, 2006):

11 $$ \mathrm{Var}\left(\hat{\boldsymbol{\uptheta}}|\boldsymbol{\uptheta} \right)=\mathbf{I}{\left(\boldsymbol{\uptheta} \right)}^{-1}=\left[\begin{array}{cc}\frac{\sum_j{s}_{j,1}^2{P}_j\left(\boldsymbol{\uptheta} \right){Q}_j\left(\boldsymbol{\uptheta} \right)}{\left|\mathbf{I}\left(\boldsymbol{\uptheta} \right)\right|}& \frac{\sum_j{s}_{j,1}{s}_{j,2}{P}_j\left(\boldsymbol{\uptheta} \right){Q}_j\left(\boldsymbol{\uptheta} \right)}{\left|\mathbf{I}\left(\boldsymbol{\uptheta} \right)\right|}\\ {}\frac{\sum_j{s}_{j,1}{s}_{j,2}{P}_j\left(\boldsymbol{\uptheta} \right){Q}_j\left(\boldsymbol{\uptheta} \right)}{\left|\mathbf{I}\left(\boldsymbol{\uptheta} \right)\right|}& \frac{\sum_j{s}_{j,2}^2{P}_j\left(\boldsymbol{\uptheta} \right){Q}_j\left(\boldsymbol{\uptheta} \right)}{\left|\mathbf{I}\left(\boldsymbol{\uptheta} \right)\right|}\end{array}\right], $$

where

12 $$ \left|\mathrm{I}\left(\boldsymbol{\uptheta} \right)\right|=\left[\sum \limits_j{s}_{j,1}^2{P}_j\left(\boldsymbol{\uptheta} \right){Q}_j\left(\boldsymbol{\uptheta} \right)\right]\left[\sum \limits_j{s}_{j,2}^2{P}_j\left(\boldsymbol{\uptheta} \right){Q}_j\left(\boldsymbol{\uptheta} \right)\right]-{\left[\sum \limits_j{s}_{j,1}{s}_{j,2}{P}_j\left(\boldsymbol{\uptheta} \right){Q}_j\left(\boldsymbol{\uptheta} \right)\right]}^2. $$

Thus, in the multidimensional case, the estimator variances cannot be directly formulated as linear functions of the decision variables (i.e., **z**). However, van der Linden (2006, p. 194) shows that variance functions can be linearly optimized by decomposing them into linear components. Specifically, the author proposes an approximation by minimizing the off-diagonal term of the information matrix and maximizing the diagonal terms by imposing lower bound constraints. This can be formulated as follows:

13 $$ \operatorname{minimize}\ {\sum}_j{z}_j{s}_{j,1}{s}_{j,2}{P}_j\left(\boldsymbol{\uptheta} \right){Q}_j\left(\boldsymbol{\uptheta} \right), $$

subject to

$$ \sum \limits_j{z}_j{s}_{j,d}^2{P}_j\left(\boldsymbol{\uptheta} \right){Q}_j\left(\boldsymbol{\uptheta} \right)\ge k,\mathrm{for}\ \mathrm{all}\ d\ \mathrm{dimensions}, $$

where several values of *k* must be iteratively tested until optimal variance functions are found. Additionally, to obtain precise **θ** estimates for each test taker, optimizing the questionnaire for a single **θ** does not suffice. Thus, to account for more than one point in the **θ** space, van der Linden (2006, p. 198) suggests the use of the multidimensional minimax approach, in which a maximum, *y*, is minimized subject to:

14 $$ \sum \limits_j{z}_j{s}_{j,1}{s}_{j,2}{P}_j\left({\boldsymbol{\uptheta}}_{\boldsymbol{l}}\right){Q}_j\left({\boldsymbol{\uptheta}}_{\boldsymbol{l}}\right)\le y,\mathrm{for}\ \mathrm{all}\ l, $$

where **θ**_***l***_ denotes a given point in an *L* × *D* quadrature grid of the selected evaluation points. Given that the TIF is a smooth, well-behaved function of **θ**, numerically approximating the TIF over a finite set of well-spread **θ** points should provide a good indicator of its true form (van der Linden, 2006).

Linear models for automated test assembly are promising, as they allow the use of general MIP solvers instead of specialized heuristics (van der Linden & Li, 2016). However, despite being an apparently straightforward solution, when applied to FCQ assembly, they can be computationally costly because of the vast size of the combinatorial search space. First, the analysis units (the decision variables) are not the items; rather, they are the feasible blocks, being that the latter are noticeably larger in number. For instance, for 60 items measuring five dimensions (12 items per dimension), there are 1440 possible hetero-dimensional item pairs. Second, the number of constraints can be substantially large. To account for a complete quadrature grid in a five-dimensional test with three quadrature points per dimension, 243 **θ** vectors must be considered (i.e., *L* = 3^5^). Therefore, to optimize the boundary of the off-diagonal elements in the information matrix (Eq. 14), 2430 linear constraints must be set (243 × 10, where 10 is the number of combinations of the five dimensions taken in twos without repetition). Furthermore, to maximize the boundary of the main diagonal elements (Eq. 13), 1215 linear constraints (243 × 5) are required. Finally, the MIP problems must be computed several times to explore Eq. 13 over a set of feasible *k*, resulting in a slow procedure that may be unrealistic with current computer processing power. To illustrate this, we conducted a preliminary study using the abovementioned questionnaire conditions and the GNU Linear Programming Kit (GLPK) solver. No convergence was found within 24 h, with a relative MIP gap of approximately 30% at that time, which is considerably higher than van der Linden and Li’s (2016) 2% compromise. Considering this, this article presents a novel approach for assembling FCQs using a genetic algorithm (GA).

## Genetic algorithm

GAs are heuristic optimization methods that search for optimal solutions through the iterative specialization of generations of individuals via mutation and selection of the fittest. Each individual (i.e., candidate solution) has a genotype code (i.e., a decision vector) that represents a phenotype (i.e., a questionnaire form). GAs are fundamentally comprised of three functional components: crossover, mutation, and selection operators. The purpose of the selection operator is to select the fittest candidates to pass on to the next generation. To accomplish this, each candidate’s fitness is evaluated based on their score in an objective function. In turn, the crossover operator generates new offspring by exchanging genotype codes between some members of the current generation. Finally, the mutation operator adds randomness to the new offspring by randomly modifying parts of the genotype code, which helps maintain the diversity within each generation and prevents premature convergence.

Among GAs, the estimation of distribution algorithms (EDAs) replaces the traditional crossover and mutation operators by sampling new candidates using probabilistic models fitted with previous generations’ genotypes. Among these algorithms, the node histogram-based sampling algorithm (NHBSA; Tsutsui, 2006) is suitable for FCQ, as it is intended to solve combinatorial problems. Specifically, in the NHBSA, the genotypes are coded as permutation vectors, where both the position and the value of each element represent a pair of entities (e.g., first and second items in a block, as will be detailed later). In this sense, new genotypes are formed in a two-step process. First, a part of the new genotype is formed by directly passing on a fraction of a parent’s genotype (referred to as template). Second, the remaining elements of the decision vector are sampled from the conditional probability distribution for the values in each element position in the decision vectors from the previous generation (which is analogous to a crossover operator). A constant error is added to the conditional probabilities as a mutation factor. After the new genotypes are formed, each candidate is compared with its parent (from which the template is inherited) in terms of constraint compliance and value in the objective function, and the better candidate from each pair continues to the next generation. The proposed adaptation of the NHBSA for forced-choice assembly is defined in more detail in the following sections.

## Decision vectors

In single-stimulus linear test assembly procedures, binary decision vectors are used to indicate whether an item is (not) selected in a test form. For assembling forced-choice pairs, however, in addition to selecting the items from a pool, the decision vector must represent how the items are paired. The decision vectors for item pairing can be efficiently represented as in the quadratic assignment problem (Koopmans & Beckmann, 1957). In the NHBSA, given an item pool with size *N*, a genotype is coded as a permutation vector **δ** = *δ*_1_, …, *δ*_*N*_, where both a given element’s value and position in **δ** are used to identify the items in a pair. Specifically, δ_*i*_ = *u* indicates that item *i* is paired with item *u*; for instance, *δ*_3_ = 7 denotes that items 3 and 7 are paired. Note that some constraints must be defined to prevent an item from being represented in multiple blocks (e.g., *δ*_3_ = 7, and *δ*_7_ = 1). As will be detailed below, in this implementation, a constraint was incorporated into the sampling operator, to ensure that the values and positions in **δ** are always symmetric (e.g., *δ*_3_ = 7 → *δ*_7_ = 3 and backwards) and so that each item is represented in a single block.

## Block content constraints

As mentioned previously, in traditional GA, compliance with the constraints is evaluated in the selection operator, where feasible (constraint compliant) candidates are favored to pass on to the next generation. When a large set of constraints must be met, only a few feasible candidates may be observed, making GA inefficient. To make it possible to optimize the FCQ with a large number of constraints, a modification was made to the original NHBSA. In the NHBSA adaptation presented here, the block content constraints are passed to the probabilistic model rather than evaluated *a posteriori*. This may lead to slower computations when sampling new decision vectors, but it brings a gain in efficiency in the long term (more feasible solutions evaluated per generation). In this sense, users may impose constraints so that blocks must fulfill certain characteristics (e.g., be formed by items assessing different traits or with similar social desirability ratings). The block content constraints are coded in a binary symmetric *N* × *N* matrix **C**, indicating whether items *i* and *u* can (*c*_*i*, *u*_ = 1) or cannot (*c*_*i*, *u*_ = 0) be paired. These constraints are considered in the probabilistic model for sampling the new genotypes. In contrast, content constraints at the questionnaire level, such as the number of items per dimension or blocks by a pair of dimensions, are set in the sampling operator, as will be presented in the following section.

## Probabilistic model

The probabilistic model used for sampling new genotypes is based on the relative frequencies of the feasible item pairs (i.e., *c*_*i*, *u*_ = 1) in the current generation, with a mutation factor added. Let **δ**^*k*, *t*^ denote the decision vector for the *k*^th^ candidate solution in the *t*^th^ generation so **δ**^*k*, *t*^ can be binarily represented as an *N* × *N* matrix **D**^*k*, *t*^ given as:

15 $$ {d}_{i,u}^{k,t}=\left\{\begin{array}{c}1\kern1.25em \mathrm{if}\ {\delta}_i^{k,t}=u\\ {}0\kern2em \mathrm{otherwise}\end{array}\right. $$

Therefore, ${\mathbf{D}}^t={\sum}_{k=1}^K{\mathbf{D}}^{k,t}$, where *K* is the user-defined population size, provides the node histogram matrix (NHM) representing the frequencies of the co-occurrence of the items in the *t*^th^ generation. The probability model for ${\delta}_i^t=u$, for *u* ∈ {1, …, *N*}, used for sampling the new mutated vectors ${\delta}_i^{k,t\ast }$ , is calculated as:

16 $$ P\left({\delta}_i^t=u|{\mathbf{c}}_{i,\cdotp },{\mathbf{d}}_{i,\cdotp}^t,{\varepsilon}_i\right)=\frac{c_{i,u}\left({d}_{i,u}^t+{\varepsilon}_i\right)}{\sum_{l=1}^N{c}_{i,l}\left({d}_{i,l}^t+{\varepsilon}_i\right)} $$

where *ε*_*i*_ denotes a mutation factor that pushes $P\left({\delta}_i^t\right)$ for all *u* toward a uniform distribution. Similar to Tsutsui (2006), *ε*_*i*_ should be proportional to the average frequency in ${\mathbf{d}}_{i,\cdotp}^t$. Given that different *i*^th^ items may have different block constraints (i.e., ${\sum}_j{c}_{i,u}$), the average frequency in ${\mathbf{d}}_{i,\cdotp}^t$is defined as $K/{\sum}_j{c}_{i,u}$, and *ε*_*i*_ is computed as:

17 $$ {\varepsilon}_i=\raisebox{1ex}{$K$}\!\left/ \!\raisebox{-1ex}{${\sum}_j{c}_{i,u}$}\right.{B}_{ratio} $$

where the bias ratio (*B*_*ratio*_) is a user-defined uniformizing positive constant controlling the mutation factor, thus setting the pace for the specialization of the probabilistic model. In other words, as can be observed in Eq. 16, as *B*_*ratio*_ → 0, thus *ε*_*i*_ → 0, the probabilistic model for sampling new genotypes leans toward the relative frequencies of the NHM in the previous generation. On the contrary, as *B*_*ratio*_ → ∞, thus *ε*_*i*_ → ∞, $P\left({\delta}_i^t\right)$ leans toward a uniform distribution. In practical terms, a higher *B*_*ratio*_ will provide greater genotype heterogeneity, reducing the risk of local optima, but it will also increase the time before convergence (i.e., when all candidate solutions in a generation have the same genotype).

## Sampling operator

**Fig. 1:**
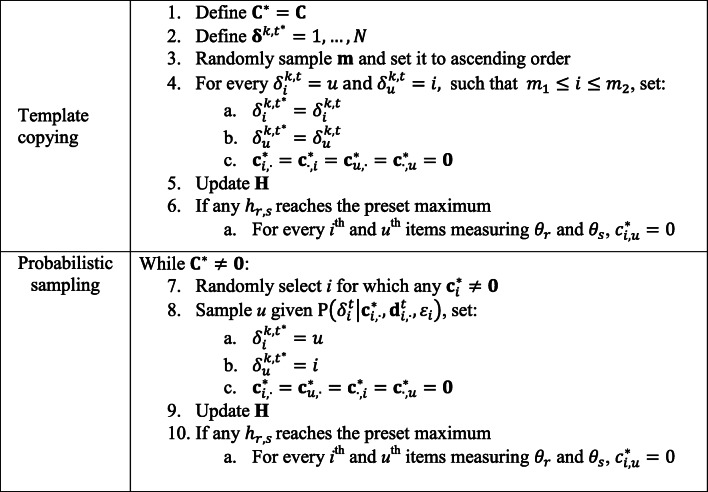
Schematic description of the sampling operator for one decision vector

Let **C**^∗^ be a temporary duplicate of the block constraint matrix **C**. Following the sampling *with template* in Tsutsui (2006), a new decision vector ${\boldsymbol{\updelta}}^{k,{t}^{\ast }}$is partly generated by copying the template of an existing vector **δ**^*k*, *t*^ and partly sampled using the probabilistic model of generation *t*. First, two ordered cut points, *m*_1_ and *m*_2_, where **m** = *m*_1_, *m*_2_ ⊂ 1, …, *N*, are randomly sampled to define the template range to be copied to the new ${\boldsymbol{\updelta}}^{k,{t}^{\ast }}$. Second, the subset including ${\delta}_i^{k,t}=u$ and ${\delta}_u^{k,t}=i$ elements such that *m*_1_ ≤ *i* ≤ *m*_2_ is passed to the new ${\boldsymbol{\updelta}}^{k,{t}^{\ast }}$ vector, whereas the remaining elements of ${\boldsymbol{\updelta}}^{k,{t}^{\ast }}$are iteratively sampled from the multinomial distribution defined by $\mathrm{P}\left({\delta}_i^t|{\mathbf{c}}_{i,\cdotp}^{\ast },{\mathbf{d}}_{i,\cdotp}^t,{\varepsilon}_i\right)$ until **C**^∗^ = **0**. Note that to maintain the symmetry of the value and position in ${\boldsymbol{\updelta}}^{k,{t}^{\ast }}$, after sampling a given *u* for ${\delta}_i^{k,{t}^{\ast }}$, the equality ${\delta}_u^{k,{t}^{\ast }}=i$ is applied. In addition, to prevent an item from appearing in two different blocks, once an element ${\delta}_u^{k,{t}^{\ast }}=i$ is fixed, all ${\mathbf{c}}_{i,\cdotp}^{\ast }$, ${\mathbf{c}}_{u,\cdotp}^{\ast }$, ${\mathbf{c}}_{\cdotp, i}^{\ast }$, and ${\mathbf{c}}_{\cdotp, u\cdotp}^{\ast }$ are set to **0**, so that, for the current candidate *k,* the items involved cannot be selected again. Furthermore, let **H** denote a *Q* × *Q* matrix of counts of blocks per pair of dimensions, where *Q* is the number of dimensions assessed via the questionnaire. If *h*_*r*, *s*_reaches a maximum preset by the user, the equality ${c}_{i,u}^{\ast }=0$ is set for every *i*^th^ and *u*^th^ item measuring *θ*_*r*_ and *θ*_*s*_. Figure 1 shows a schematic of the sampling operator.

If **C**^∗^ = **0** and the equality ${\delta}_i^{k,{t}^{\ast }}=i$ exists for any *i* ∈ 1, …, *N* (meaning that the *i*^th^ element has not been modified from the initial ${\boldsymbol{\updelta}}^{k,{t}^{\ast }}$), it indicates that the *i*^th^ item is paired with itself, which implies that it is left out of the questionnaire for calculating the objective function. This will occur when the number of items included in the questionnaire is lower than the total item pool size (i.e., 2 *J* < *N*).

## Evaluation and selection

After sampling the *K* new candidate genotypes, the objective function *obj*(·), is calculated for each phenotype of ${\boldsymbol{\updelta}}^{k,{t}^{\ast }}$. In contrast to the original NHBSA, in this implementation, each candidate is not compared only with its parent, that is, *obj*(**δ**^*k*, *t*^) vs. *obj*(${\boldsymbol{\updelta}}^{k,{t}^{\ast }}$); rather, all candidates in *t* and t^*^ are compared. The decision vectors associated with the *K* best *obj*(·) (i.e., the highest for maximization and the lowest for minimization problems) in the union of *t* and t^*^ are selected to constitute the population in *t* + 1. As mentioned above, single-item blocks (i.e., ${\delta}_i^{k,{t}^{\ast }}=i$) are omitted from the calculation of the objective functions.

## Main loop

**Fig. 2:**
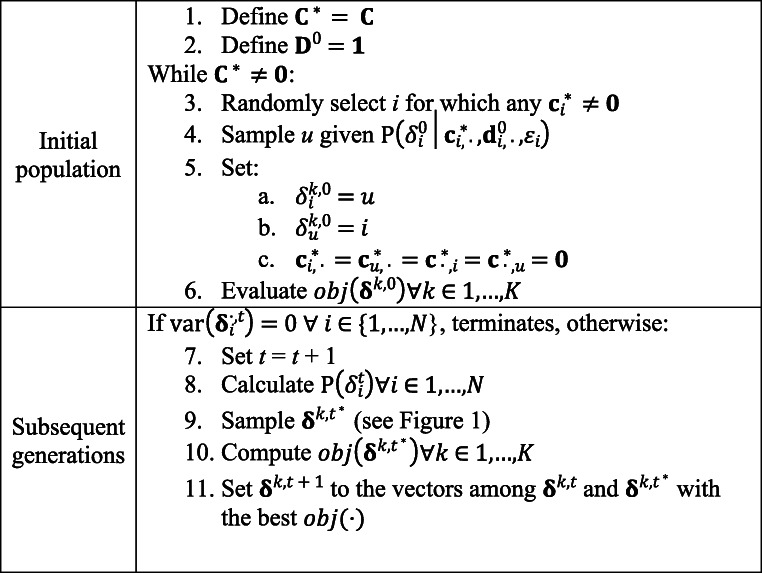
Schematic description of the main loop

The main loop of the forced-choice block assembly algorithm is schematically represented in Fig. 2. As indicated, the initial population is randomly sampled with uniform $P\left({\delta}_i^0=u|{\mathbf{c}}_{i,\cdotp}^{\ast },{\mathbf{d}}_{i,\cdotp}^0,{\varepsilon}_i\right)$ for every *u* satisfying ${\mathbf{c}}_{i,u}^{\ast }=1$, where **C**^∗^ is initiated as the user-defined binary matrix representing the constraints on the block contents, as in the sampling operator. The algorithm runs until all *K* candidate solutions within a population have the same decision vectors (i.e., $\mathit{\operatorname{var}}\left({\boldsymbol{\updelta}}_i^{\cdotp, t}\right)=0\forall i\in 1,\dots, N$).

## Method

A simulation study was conducted to evaluate the performance of the proposed GA for forced-choice item pairing. To compare its efficiency, a quasi-exhaustive brute-force (BF) search was carried out, with its runtime matched to the time for the convergence of the GA under each respective simulation condition. In addition, as will be further detailed, a set of trait score recovery criteria was calculated for each generated questionnaire.

The candidate questionnaires obtained through the quasi-BF search allowed for the establishment of two benchmarks. First, the average of the trait recovery criteria across the candidate questionnaires in each condition served as an indicator of the expected accuracy of a randomly assembled questionnaire for a given item pool. This indicator aims to represent the results for FCQs built using some structural criteria (e.g., number of items per dimension), which is a common practice in current research involving the forced-choice format (e.g., Bürkner et al., 2019; Walton et al., 2020). Second, the questionnaire with the highest objective function value among the candidates served as an indicator of the best accuracy obtained using an alternative heuristic procedure. By matching the computation times in the BF search to the GA, the two methods were compared in terms of efficiency. Both procedures were executed with a 3.60 GHz Intel® Core™ i7-4790 CPU and 16.00 RAM using the MS Windows 7 Professional operating system.

### Data generation

Five-dimensional item pools with an equal number of items per dimension were simulated to emulate the real problem of generating a forced-choice personality questionnaire under the five-factor model (Costa & McCrae, 1992). When defining the FCQ design, two real case scenarios were considered: (1) forming blocks using all the items in the pool and thus only pairing the items, and (2) assembling a questionnaire using only part of the item pool, which involves both selecting and pairing the items. Accordingly, the two chosen ratios of item pool size to FCQ length were *N*:*J* = 2:1 (pairing all items) and *N*:*J* = 8:1 (selecting and pairing a quarter of the items). Two FCQ lengths were defined: *J* = 30 (i.e., six blocks per dimension) and *J* = 60 (i.e., 12 blocks per dimension). To achieve the aforementioned *N*:*J* ratios for these FCQ lengths (*J* = 30 and *J* = 60), item pool sizes (*N*) of 60 and 240 items, and 120 and 480 items, respectively, were generated.

For each item pool, the discrimination parameters (*a*_*j*_) were sampled from an *N*(1.5, 0.5) distribution and item difficulty parameters (*b*_*j*_) from a *U*(−2.0, 2.0) distribution. The distribution of discrimination parameters was chosen to make negatively keyed items very unlikely. Finally, as in Brown and Maydeu-Olivares (2011), the true latent trait correlation matrix (**Φ**) was set as either a five-dimensional identity matrix (***I***_5_) or as the one observed for the revised NEO personality inventory (NEO PI-R; Costa & McCrae, 1992) with empirical data (see Table 1). Twenty item pools were generated for each condition.

**Table 1:** Trait correlation matrix observed in the NEO PI-R (Costa & McCrae, 1992) with neuroticism reversed to emotional stability

	ES	EX	OE	AG	CO
ES	1				
EX	0.21	1			
OE	0	0.4	1		
AG	0.25	0	0	1	
CO	0.53	0.27	0	0.24	1

*Note*. ES: emotional stability, EX: extraversion, OE: openness to experiences, AG: agreeableness, CO: conscientiousness.

To analyze the recovery of the trait estimates, as will be further detailed, the true trait scores ~*MVN*(**0**, **Φ**) were generated for 1000 simulees and for each simulated item pool. Forced-choice response data were then sampled given the probabilities under the MUPP-2PL model using the true item parameters of each FCQ analyzed in each condition.

### Assembly procedure specifications

#### Questionnaire constraints

For both GA and BF searches, the constraints were set as follows: (1) each FCQ had the exact designed length (i.e., *J* = 30 or *J* = 60), (2) each item could only be assigned to one block, (3) the items in each block addressed different dimensions, and (4) the number of blocks measuring each pair of dimensions was the same.

### Objective function

The objective function to maximize was the average of the posterior marginal reliabilities (${\hat{\rho}}_{\theta {\hat{\theta}}_d}^2$) across the five dimensions, calculated using the marginal posterior error variances (Eq. 18). The average of ${\hat{\rho}}_{\theta {\hat{\theta}}_d}^2$ over *d* = {1, …, *D*} is an intuitive objective function for applied researchers and is inversely proportional to the widely used A-optimality criterion:

18 $$ {\displaystyle \begin{array}{c}{\overline{\operatorname{var}}}_d\left(\hat{\boldsymbol{\uptheta}}|\boldsymbol{\uptheta} \right)={\sum}_{l=1}^L{\left[\mathbf{I}\left({\boldsymbol{\uptheta}}_l\right)+{\boldsymbol{\Phi}}^{-\mathbf{1}}\right]}_{d,l}^{-\mathbf{1}}\cdotp g\left({\boldsymbol{\uptheta}}_l|\boldsymbol{\Phi} \right),\mathrm{and}\\ {}{\hat{\rho}}_{\theta {\hat{\theta}}_d}^2=1-{\overline{\operatorname{var}}}_d\left(\hat{\boldsymbol{\uptheta}}|\boldsymbol{\uptheta} \right),\end{array}} $$

where *l* represents each possible combination of the quadrature points {−2, 0, +2} over each of the *D* (i.e., 5) dimensions; thus, *L* = 3^5^, and *d* denotes the *d*^th^ diagonal element of each matrix associated with the *d*^th^ dimension of **θ**_*l*_. The terms [**I**(**θ**_*l*_) + **Φ**^***−*1**^] and *g*(**θ**_*l*_| **Φ**) correspond to the posterior information matrix at quadrature point *l*, respectively, and the multivariate normal density function at each **θ**_*l*_ is given the true **Φ** correlation matrix. The use of posterior information matrices instead of Fisher information matrices, analogous to Segall (1996), improves the efficiency of Bayesian estimates by accounting for the prior trait variance-covariance matrix.

#### Genetic algorithm specifications

User-defined specifications for the NHBSA are each generation’s population size and the bias ratio constant (i.e., the mutation factor). The decision on population sizes in the NHBSA reflects a balance between two important factors: (1) the precision of the probabilistic model for sampling decision vectors and (2) the computation time. The larger the population size, the better the node histograms’ approximation of the probabilistic model, but the slower the algorithm will be. In contrast, the *B*_*ratio*_ sets the amount of mutation in the probability model. On the one hand, as *B*_*ratio*_ increases, NHBSA probabilistic models tend toward a uniform distribution, approaching performance comparable with the BF search. On the other hand, if *B*_*ratio*_ is zero, no mutation is added to the probability model, and only the blocks included in the initial population are considered. In the present study, the population size was set as equal to the item pool size (*K* = *N*), and the *B*_*ratio*_ was set to 2^−4^, as in Tsutsui (2006). Finally, as shown in Fig. 2, the algorithm was considered to have converged whenever all the candidate solutions within a generation had the same genotype (i.e., $\mathbf{\operatorname{var}}\left({\boldsymbol{\updelta}}_i^t\right)=0\forall i\in \left\{1,\dots, N\right\}$).

#### Brute-force search specifications

A stepwise constrained random sampling procedure was conducted to fulfill the content constraints, as in the probabilistic sampling procedure outlined in Fig. 1, with the exception that the values for $\mathrm{P}\left({\delta}_i^t|{\mathbf{c}}_{i,\cdotp}^{\ast },{\mathbf{d}}_{i,\cdotp}^t,{\varepsilon}_i\right)$ were uniformly distributed. As in any BF search, this procedure was carried out multiple times for each item pool condition and replication, yielding a considerable number of candidate questionnaires. On average, approximately 113,845 candidate questionnaires were evaluated for each simulation condition and replication in the BF search.

### Comparison criteria

The quality of the questionnaires obtained through each assembly procedure was assessed through the recovery of the maximum *a posteriori* (MAP) scores from simulated response datasets, estimated using the *mirt* package (Chalmers, 2012) in *R* software (R Core Team, 2020) with block parameters fixed to their true values. The criteria for the recovery of the trait estimates were: (1) the average true reliability, calculated using the squared correlation between true and estimated *θ* (${\rho}_{\theta \hat{\theta}}^2$); (2) the average root mean square error between the estimated and true *θ* (${\mathrm{RMSE}}_{\hat{\theta}}$); (3) the average trait correlation bias (${\mathrm{Bias}}_{\hat{\boldsymbol{\Phi}}}$); and (4) the average ${\mathrm{RMSE}}_{\hat{\theta}}$, bias, and standard error of $\hat{\theta}$ conditional to the true *θ*. ${\mathrm{RMSE}}_{\hat{\theta}}$ and ${\mathrm{Bias}}_{\hat{\boldsymbol{\Phi}}}$ were computed as in Equations 19 and 20, respectively:

19 $$ {\mathrm{RMSE}}_{\hat{\theta}}=\sqrt{\sum \limits_{s=1}^S\frac{{\left({\hat{\theta}}_s-{\theta}_s\right)}^2}{S}} $$

20 $$ {\mathrm{Bias}}_{\hat{\boldsymbol{\Phi}}}=\hat{\boldsymbol{\Phi}}-\boldsymbol{\Phi}, $$

where *S* is the total number of simulees (i.e., *S* = 1000), and parameters $\hat{\boldsymbol{\Phi}}$ and **Φ** are the estimated and true trait correlation matrices, respectively. The true reliability and the ${\mathrm{RMSE}}_{\hat{\theta}}$ were computed for each dimension separately and then averaged across the five traits, whereas the ${\mathrm{Bias}}_{\hat{\boldsymbol{\Phi}}}$ was calculated by averaging the Fisher Z-transformed differences of the non-diagonal elements of **Φ** and backtransforming the average to the correlation metric (e.g., Corey et al., 1998). To synthesize the results, mixed-effects analyses of variance (ANOVAs) were conducted to evaluate the effect of the assembly method (within-group factor), the number of blocks, and the items-to-blocks ratios (between-group factors) on the trait estimate recovery indicators. Generalized eta-squared (Olejnik & Algina, 2003) effect sizes are presented to describe the relevance of the effects. All analyses were conducted using *R* software (R Core Team, 2020) and mixed-effect ANOVAs were performed with the Type III sum of squares using the *afex* package (Singmann et al., 2020).

## Results

### Algorithm efficiency

All GA trials converged within a reasonable time, with averages of 0.77 and 5.07 minutes in the 30-block condition with 2:1 and 8:1 *N*:*J* ratios, respectively, and 18.47 and 181.42 minutes in the 60-block condition with 2:1 and 8:1 *N*:*J* ratios, respectively. As already mentioned in the Method section, the BF search was bounded to the convergence times obtained with the GA, as an exhaustive BF search is unfeasible.

**Fig. 3:**
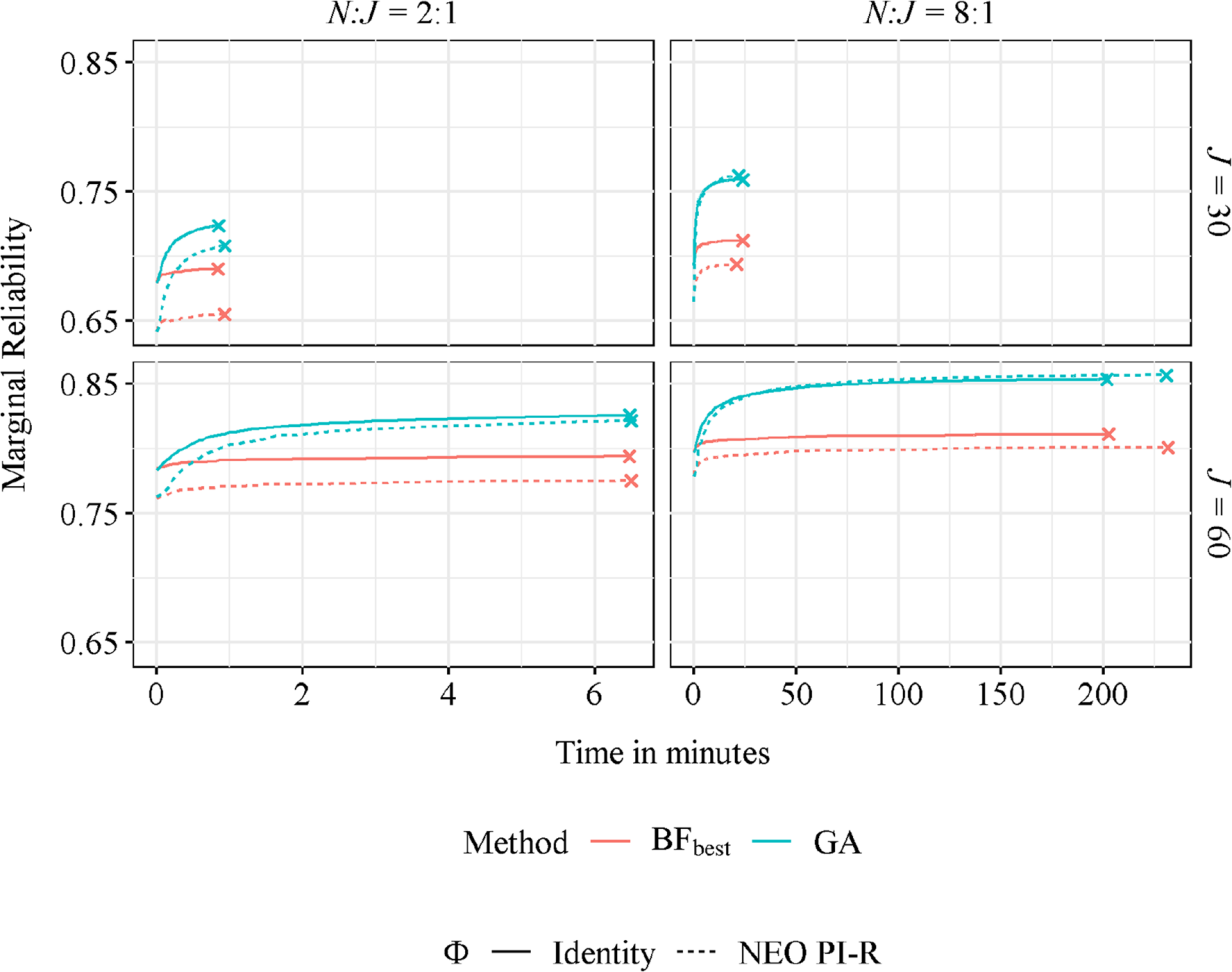
Average posterior marginal reliability over time for the best candidates in the genetic algorithm and a brute-force search. *Note*. *J* = number of blocks; *N*:*J* ratio = items-to-block ratio.

Figure 3 represents the progress of the best questionnaires’ objective function (i.e., average posterior marginal reliability) over time with the GA and BF search. As can be observed, although the initial solutions were similar with both procedures, the GA rapidly overtook the best candidates formed with the constrained random assembly in the BF search. Furthermore, the BF search presents very small improvement rates, suggesting that it would take a long time to reach the results obtained through the GA.

### Recovery of trait parameters

The values of the trait recovery indicators are listed in Table 2. As expected, when matched by time, the trait recovery for questionnaires formed with the GA consistently outperformed the best of those formed through the BF search (i.e., BF_best_). In general, the true reliabilities of the GA-assembled questionnaires were found to be acceptable even in the worst simulated condition (i.e., pairing 30 blocks from a 60-item pool with NEO PI-R correlations). In an actual individual assessment, however, more than 30 blocks are recommended to achieve reasonable measurement accuracy. The average biases of the trait correlation matrices were slightly negative under all conditions, as an indicator of remnant *ipsativity,* and they became closer to zero under the conditions with uncorrelated traits as well as with the increment of questionnaire length and items-to-blocks ratio. The average estimate recovery with the BF search (i.e., BF_avg_) was considerably worse than both BF_best_ and GA under all conditions. Such results draw attention to what may be expected for questionnaires assembled using only structural criteria, such as the number of items per dimension.

**Table 2:** Average trait recovery across 20 replications for questionnaires assembled using the genetic algorithm and a brute-force search

Φ	*J*	*N*:*J* Ratio	${\rho}_{\theta \hat{\theta}}^2$			${\mathrm{RMSE}}_{\hat{\theta}}$			${\mathrm{Bias}}_{\hat{\boldsymbol{\Phi}}}$		
			GA	BF_best_	BF_avg_	GA	BF_best_	BF_avg_	GA	BF_best_	BF_avg_
Identity	30	2	0.72	0.68	0.65	0.54	0.57	0.59	−0.10	−0.12	−0.14
		8	0.75	0.70	0.65	0.51	0.55	0.59	−0.07	−0.11	−0.14
	60	2	0.82	0.79	0.76	0.43	0.46	0.49	−0.08	−0.10	−0.12
		8	0.84	0.80	0.76	0.40	0.44	0.49	−0.05	−0.08	−0.12
NEO PI-R	30	2	0.69	0.65	0.59	0.56	0.60	0.64	−0.13	−0.16	−0.20
		8	0.74	0.68	0.59	0.51	0.57	0.64	−0.07	−0.12	−0.20
	60	2	0.81	0.77	0.73	0.44	0.48	0.52	−0.08	−0.11	−0.15
		8	0.84	0.79	0.73	0.40	0.46	0.52	−0.04	−0.09	−0.15

*Note*. **Φ** = true trait correlation matrix; *J* = number of blocks; *N*:*J* ratio = items-to-blocks ratio; ${\rho}_{\theta \hat{\theta}}^2$= true reliability; ${\mathrm{RMSE}}_{\hat{\theta}}$ = root mean square error; ${\mathrm{Bias}}_{\hat{\boldsymbol{\Phi}}}$ = trait correlation bias; GA: genetic algorithm; BF_best_ = best brute-force solution; BF_avg_ = average of brute-force solutions. The standard deviations of the indicators across replications ranged from 0.003 to 0.016.

The ANOVA effect sizes are presented in Table 3. It can be observed that the accuracy of the latent trait estimates (i.e., ${\rho}_{\theta \hat{\theta}}^2$ and ${\mathrm{RMSE}}_{\hat{\theta}}$) was mainly affected by the number of blocks (i.e., *J*), followed by the assembly method, the generated trait correlation matrix (i.e., **Φ**), and the *N*:*J* ratio. In addition, large effect sizes (i.e., ${\eta}_G^2\ge 0.14$) were found for the two-way interactions of the assembly method with **Φ** and with the *N*:*J* ratio. The first interaction effect indicates that the improvement seen by using the GA as opposed to the BF search solutions (i.e., BF_best_ and BF_avg_) was substantially higher when the traits being measured were positively correlated. The second interaction indicates that when the assembly condition required selecting items in addition to pairing them (i.e., *N*:*J* ratio = 8:1), the GA was notably more effective than the BF search. Similarly, the biases of the correlation estimates were higher when the traits were correlated (i.e., **Φ** from the NEO PI-R), the test length was short, and there was no possibility of selecting items (i.e., *N*:*J* = 2). However, it should be noted that the assembly method had a greater effect size, indicating that assembling the questionnaires properly can be even more effective than making them longer or using greater item pools and can substantially attenuate the *ipsativity* inherent in positively correlated traits. Three- and four-way interactions offered small effect sizes in all three comparison criteria.

**Table 3:** Generalized eta-squared effect sizes for mixed-effects ANOVAs of trait estimate recovery indicators

	${\rho}_{\theta \hat{\theta}}^2$	${\mathrm{RMSE}}_{\hat{\theta}}$	${\mathrm{Bias}}_{\hat{\boldsymbol{\Phi}}}$
**Within-group effects**			
Method	0.91^**^	0.91^**^	0.81^**^
Method × *J*	0.12^**^	0.01^*^	0.01^*^
Method × *N*:*J* Ratio	0.28^**^	0.32^**^	0.21^**^
Method × **Φ**	0.31^**^	0.27^**^	0.28^**^
Method × *J* × *N*:*J* Ratio	0.01^*^	0.00	0.01^*^
Method × *J* × **Φ**	0.02^**^	0.00	0.03^**^
Method × *N*:*J* Ratio × **Φ**	0.02^**^	0.02^**^	0.02^**^
Method × *J* × *N*:*J* Ratio × **Φ**	0.00	0.00	0.00
**Between-group effects**			
*J*	0.96^**^	0.96^**^	0.58^**^
*N*:*J* Ratio	0.40^**^	0.43^**^	0.30^**^
**Φ**	0.54^**^	0.51^**^	0.41^**^
*J* × *N*:*J* Ratio	0.01	0.00	0.01
*J* × **Φ**	0.09^**^	0.04^*^	0.11^**^
*N*:*J* Ratio *×* **Φ**	0.02^*^	0.02^*^	0.03^*^
*J* × *N*:*J* Ratio × **Φ**	0.01	0.00	0.01

*Note*. *J* = number of blocks; *N*:*J* ratio = items-to-blocks ratio; ${\rho}_{\hat{\theta}\theta}^2$ = true reliability; **Φ** = true trait correlation matrix; ${\mathrm{RMSE}}_{\hat{\theta}}$ = root mean square error; ${\mathrm{Bias}}_{\hat{\boldsymbol{\Phi}}}$= trait correlation bias; ^*^ *p* < 0.05; ^**^ *p* < 0.001.

### Conditional estimation errors

**Fig. 4:**
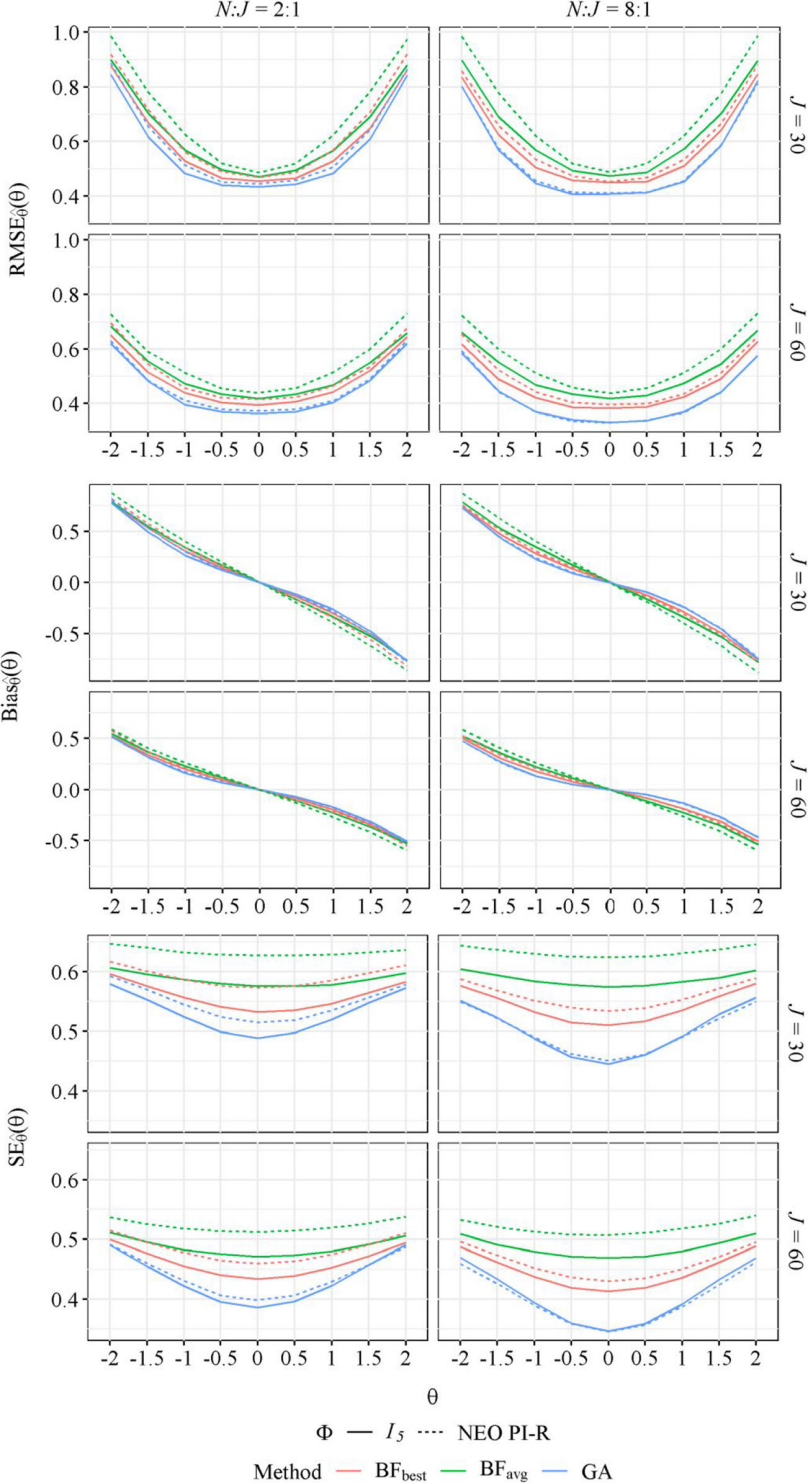
Average conditional RMSE, bias, and standard errors of estimates for different assembly methods and true trait correlation matrices (**Φ**)

As shown in Fig. 4, the questionnaires assembled with the GA showed the recovery of *θ* in all simulation conditions and throughout the *θ* continuum. In accordance with the reliability results, the effect of the assembly method increased as the *N*:*J* ratio increased. As can be inferred from the conditional ${Bias}_{\hat{\theta}}$ results, the distribution of $\hat{\theta}$ was compressed toward the mean, as is characteristic of Bayesian estimators. In addition, as can be expected from the definition of the objective function in Eq. 18, the recovery of *θ* was best for those *θ* closer to 0, as a higher weight is given to the conditional error variances for *θ* → 0 in the calculation of the marginal posterior reliabilities. Finally, as expected, under the conditions with the generated NEO PI-R correlation matrix (dotted lines in Fig. 4), the distributions of estimation errors were similar or worse than under the conditions with independent traits (solid lines).

## Follow-up study

As discussed in the Introduction section, the decision to include opposite-keyed item pairs is still unclear. On the one hand, as Bürkner et al. (2019) have argued, questionnaires including hetero-polar blocks may be intuitively understood as less robust to SDR and faking in certain situations, as respondents may easily identify and select the most desirable option in a block. On the other hand, questionnaires including only positively keyed items may have remnant *ipsativity*, as suggested by the negatively biased trait intercorrelations found in the previous study, and lower precision of trait estimates (e.g., Brown & Maydeu-Olivares, 2011; Bürkner et al., 2019). Thus, a second simulation study was conducted to investigate the performance of the proposed GA for forced-choice pairing, including hetero-polar blocks.

This study replicated the two chosen ratios of item pool size to FCQ length (*N*:*J* = 2:1 and *N*:*J* = 8:1) and the two FCQ lengths (*J* = 30 and *J* = 60). As in Brown and Maydeu-Olivares (2011), the FCQs were constrained to having one half consisting of homo-polar blocks and the other half consisting of hetero-polar blocks. Therefore, to fulfill this constraint, item pools were simulated with a quarter of negatively keyed items, which is the proportion of negative items in the final constraint-compliant questionnaires. The discrimination parameters of the negatively keyed items were sampled from an *N*(−1.5, 0.5) distribution, and all other parameters were replicated from the previous study. In addition, as in the previous study, the recovery of trait estimates was assessed by the true reliability, the ${\mathrm{RMSE}}_{\hat{\theta}}$, the trait correlation bias, and the average conditional standard error, and the results were compared with a quasi-exhaustive BF search.

The results for the trait recovery indicators with the FCQ composed of one half homo-polar blocks and the other half hetero-polar blocks are presented in Table 4. Compared to the questionnaires with all homo-polar blocks in the previous study (Table 2), all *θ* recovery indicators were better. On the one hand, due to the inclusion of hetero-polar blocks, the average trait correlation bias was especially reduced, regardless of the assembly method (${\eta}_G^2=0.02$ for the assembly method factor). In addition, **Φ** had a smaller impact on the recovery of the person parameters (${\eta}_G^2=0.20$, ${\eta}_G^2=0.22$, and ${\eta}_G^2=0.13$ for ${\rho}_{\theta \hat{\theta}}^2$, ${\mathrm{RMSE}}_{\hat{\theta}}$ and ${\mathrm{Bias}}_{\hat{\boldsymbol{\Phi}}}$, respectively) compared with the FCQ with all homo-polar blocks. On the other hand, although this general improvement was observed in all indicators, using the GA still provided substantial gains in ${\rho}_{\theta \hat{\theta}}^2$ and ${\mathrm{RMSE}}_{\hat{\theta}}$ (${\eta}_G^2=0.86$ and ${\eta}_G^2=0.87$ for assembly method over ${\rho}_{\theta \hat{\theta}}^2$ and ${\mathrm{RMSE}}_{\hat{\theta}}$, respectively).

**Table 4:** Average trait recovery across 20 replications for questionnaires assembled using the genetic algorithm and a brute-force search with one half consisting of hetero-polar blocks

**Φ**	*J*	*N*:*J* Ratio	${\rho}_{\theta \hat{\theta}}^2$			${\mathrm{RMSE}}_{\hat{\theta}}$			${\mathrm{Bias}}_{\hat{\boldsymbol{\Phi}}}$		
			GA	BF_best_	BF_avg_	GA	BF_best_	BF_avg_	GA	BF_best_	BF_avg_
Identity	30	2	0.77	0.75	0.71	0.49	0.50	0.54	0.00	0.00	0.00
		8	0.81	0.77	0.72	0.45	0.48	0.53	0.00	0.00	0.00
	60	2	0.87	0.86	0.84	0.37	0.38	0.40	0.00	0.00	0.00
		8	0.89	0.87	0.84	0.34	0.37	0.40	0.00	0.00	0.00
NEO PI-R	30	2	0.78	0.76	0.73	0.48	0.49	0.52	0.01	0.01	0.02
		8	0.82	0.78	0.74	0.44	0.47	0.51	0.01	0.01	0.01
	60	2	0.87	0.86	0.85	0.36	0.37	0.39	0.00	0.01	0.01
		8	0.89	0.87	0.85	0.33	0.36	0.39	0.00	0.01	0.01

*Note*. **Φ** = true trait correlation matrix; *J* = number of blocks; *N*:*J* ratio = items-to-blocks ratio; ${\rho}_{\theta \hat{\theta}}^2$= true reliability; ${\mathrm{RMSE}}_{\hat{\theta}}$ = root mean square error; ${\mathrm{Bias}}_{\hat{\boldsymbol{\Phi}}}$ = trait correlation bias; GA: genetic algorithm; BF_best_ = best brute-force solution; BF_avg_ = average of brute-force solutions. The standard deviations across the replications ranged from 0.004 to 0.026.

## Discussion

The precision of trait estimates has been thoroughly pointed out as a main weakness in the use of FCQs (Brown & Maydeu-Olivares, 2011; Kreitchmann et al., 2019; Meade, 2004; Wetzel et al., 2020). Accordingly, the aim of this study was to investigate the effect of the assembly of pairwise FCQs on the recovery of trait estimates, presenting an efficient automated optimization procedure. In general, it has been shown that a single-stimulus item pool can lead to FCQs with very different psychometric properties. Therefore, naively pairing blocks without accounting for psychometric criteria can lead to suboptimal questionnaires that do not take full advantage of items’ potential. Accordingly, researchers are advised to take special care when comparing reliabilities and validities between different response formats (e.g., single-stimulus, forced-choice pairs, triplets, quads), as sub-optimally assembled questionnaires can provide less accurate trait estimates, thus lowering the upper boundary of validities. In this sense, in both scenarios, i.e., with and without hetero-polar blocks, the proposed GA appears to be an effective solution, offering substantially better trait estimates than a quasi-BF search within a reasonable time. Such improvements were especially important when the traits were positively correlated with each other. When using only homo-polar blocks, however, the trait correlations were, on average, negatively biased, indicating that some *ipsativity* remained, and this was not entirely controlled by assembling FCQs with the GA. Questionnaires including hetero-polar blocks did not have this problem and are recommended whenever SDR and faking are not expected. When exclusively using homo-polar blocks, designing longer questionnaires and optimizing them with the GA was shown to reduce remnant *ipsativity*. In addition, although the negative intercorrelation biases were considerably smaller than what would be expected for completely *ipsative* sum scores, that is, an expected average correlation of −1/(*D* − 1) =  − 0.25, the effect of remnant *ipsativity* in the correlations with external variables was not investigated. In this sense, as indicated by Hicks (1970), the sum of the correlations between the completely *ipsative* scores measured by the FCQ and each external criterion is zero. Therefore, future studies may consider addressing the relationship between homo-polar-only FCQ scores and external variables to investigate how remnant *ipsativity* affects convergent/discriminant and criterion validities.

It should be noted that the current convergence criterion of the proposed GA (i.e., achieving a unique solution in a generation) might be considered too strict, as the true reliabilities remain somewhat stable for a considerable time before convergence. In real-time implementation, it is possible to reduce the runtime by stopping the heuristics whenever a certain desired degree of stability in the objective functions is achieved.

Some caveats related to this study are acknowledged. First, as van der Linden and Li (2016) have pointed out, an important drawback of GAs is their lack of generalizability to other problems. One limitation of the presented procedure is that it was only conceived to form pairwise FCQs. Although it is possible to adapt the definitions of the node histogram and constraint matrices to greater dimensionalities (i.e., more items per block), we restricted the study to the most basic format. However, this is a very popular format in the current literature (e.g., Bunji & Okada, 2020). Nonetheless, given the potential reliability gain to be had by using triplets instead of pairs (Joo et al., 2020), a future study could attempt to extend this method to other formats. Second, in the present simulation study, we did not consider the items’ social desirability. Note, however, that it could be easily incorporated into the GA, either by setting zeros in the block constraint matrix **C** for those pairs that are not matched in social desirability or accounting for it in the IRT model. A third caveat is related to the fact that the posterior marginal reliabilities were calculated using the single-stimulus item parameters, assuming they were invariant in the forced-choice format. Nonetheless, this might not be problematic, as there is already some evidence about the invariance across formats (Lin & Brown, 2017; Morillo et al., 2019).

Finally, it should be noted that assembling questionnaires based uniquely on content constraints or item characteristics such as social desirability ratings or keyed direction may serve to increase robustness against social desirability responding and faking in high-stakes assessments (Cao & Drasgow, 2019). However, it does not guarantee the best possible trait score accuracy. Accordingly, aiming to promote best practices in the assembly of pairwise FCQs, a user-friendly implementation of the presented GA has been made available at https://psychometricmodelling.shinyapps.io/FCoptimization/. In addition, R codes can be made available upon request to the corresponding author.
